# The Bedding and Linen Department.—II

**Published:** 1908-05-16

**Authors:** 


### Nursing Administration.

### THE BEDDING AND LINEN DEPARTMENT.

### III.—THE QUANTITIES REQUIRED.

It is impossible to lay down any hard-and-fast rule as to the amount of linen required in any given hospital. Attempts have been often made to construct an inventory of necessary hospital linen from an abstract point of view, but they all fail when it comes to put them in practice. There are so many variations in custom, such wide differences as between the routine of one hospital and another, that even the most lavish estimates of what is required would need revising after a few weeks' experience, and every hospital must in effect have its own standard of efficiency in the linen department. Yet in no particular is mutual comparison more helpful. The class of work must be taken into consideration as well as the conditions under which the work is carried on. The ordinary general hospital in town or country, with no medical school and no special pressure on the beds, can work on a close margin of supplies without experiencing more than occasional inconvenience in some sudden emergency. When the cases linger on in hospital until distinctly convalescent and, as in Poor-law infirmaries, many of them are almost of a chronic type, the demands made upon the linen cupboard are easily coped with. For such hospitals the following estimate is a reasonable one, only the more ordinary articles in use being specified:

| Per Bed. | Per Ward (24 to 30 beds). |
|---|---|
| 4 sheets | 24 doctors' towels |
| 3 draw sheets | 12 basin cloths |
| 3 blankets | 12 bedpan cloths |
| 1 counterpane or quilt | 12 teacloths |
| 3 pillowcases | 12 roller towels |
| | 12 dusters * |

If we take the actual list of linen used in a clinical hospital we shall find not only that the quantities needed of the specified articles are largely in excess of the above estimate, but also that the list is open to almost indefinite expansion. The following inventory of the linen equipment of a ward of thirty beds in one of the smaller clinical hospitals reveals the difference:

| | |
|---|---|
| 72 counterpanes | 36 glass cloths |
| 140 blankets | 120 dusters |
| 8 red blankets (large) | 6 wringers |
| 4 red blankets (small) | 18 flannel operating gowns |
| 180 sheets | 48 white bedpan covers |
| 180 draw sheets | 8 blue bedpan covers |
| 180 pillowcases | 72 basin cloths, red stripe |
| 120 doctors' towels | 48 pan cloths, blue stripe |
| 36 round towels | 36 hot-water bottle covers |
| 102 teacloths | |

It will be seen that this is a very large estimate compared with the first named, which, we understand, was based on the requirements of a non-clinical hospital, where very careful management was exercised. The number of sheets and pillow-cases per bed rises to six, and in the ward stock of towels and cloths the discrepancy is even more striking. Where the clinical ward needs 120 doctors' towels the non-clinical ward finds twenty-four sufficient, it being, however, well understood that these figures do not represent the actual number of articles used in a week, but merely the stock it is found necessary to have at hand.

We are permitted to quote one more list, giving the requirements for medical and surgical wards respectively in a large clinical hospital:

| Articles Provided. | Women's Medical Ward. 44 beds. | Men's Surgical Ward. 22 beds. |
|---|---|---|
| Doctors' overalls ... ... ... | 6 | 4 |
| Bags, book ... ... ... ... | 55 | 35 |
| Bags, brush ... ... ... ... | 55 | 35 |
| Bags, hot water ... ... ... | 20 | 12 |
| Bedtops ... ... ... ... | 3 | 5 |
| Blankets ... ... ... ... | 160 | 88 |
| Blankets, cot ... ... ... | 12 | 12 |
| Blankets, foot ... ... ... | 20 | 4 |
| Pillowcases ... ... ... | 150 | 80 |
| Cloths, coarse ... ... ... | 36 | 22 |
| Cloths, glass ... ... ... | 40 | 47 |
| Cloths, locker ... ... ... | 88 | 58 |
| Cloths, pan ... ... ... | 40 | 20 |
| Cloths, table ... ... ... | 12 | 12 |
| Cloths, check ... ... ... | 12 | 8 |
| Counterpanes ... ... ... | 55 | 38 |
| Counterpanes, cot ... ... | 6 | 3 |
| Covers, sandbag ... ... | 4 | 26 |
| Covers, screen ... ... ... | 14 | 14 |
| Covers, mackintosh stand ... | 4 | — |
| Curtains ... ... ... ... | 118 | 52 |
| Curtains, cot ... ... ... | 6 | 3 |
| Dusters ... ... ... ... | 50 | 40 |
| Rubbers ... ... ... ... | 50 | 40 |
| Gowns, women's dressing ... | 14 | — |
| Gowns, girls' dressing ... ... | 6 | — |
| Gowns, flannel ... ... ... | 26 | 60 |
| Gowns, night, large ... ... | 130 | — |
| Gowns, night, medium... ... | 30 | — |
| Jackets ... ... ... ... | 50 | 30 |
| Mattresses ... ... ... | 45 | 23 |
| Mackintoshes, long ... ... | 6 | 5 |
| Mackintoshes, small ... ... | 36 | 14 |
| Mackintoshes, dressing ... | — | 7 |
| Rugs, woollen ... ... ... | 12 | 10 |
| Sheets ... ... ... ... | 200 | 118 |
| Sheets, cot ... ... ... | 12 | 6 |
| Sheets, draw ... ... ... | 200 | 96 |
| Sheets, mortuary ... ... | 10 | 6 |
| Sundries ... ... ... ... | 72 | 30 |
| Ticks, pillow ... ... ... | 24 | 46 |
| Sandbags ... ... ... | 6 | 26 |
| Towels, operation ... ... | 3 | 14 |
| Towels, operation, small ... | 12 | 30 |
| Towels, patients' ... ... | 84 | 46 |
| Towels, huckaback ... ... | 50 | 60 |
| Towels, round ... ... ... | 30 | 20 |
| Towels, wringers ... ... | 12 | 6 |
| Towels, special ... ... ... | — | 12 |
| Brushes ... ... ... ... | 42 | 22 |
| Combs ... ... ... ... | 42 | 22 |
| Dressing coats ... ... ... | — | 6 |
| Cushion covers ... ... ... | — | 24 |
| Tent ... ... ... ... | — | 1 |
| Shirts, men's ... ... ... | — | 70 |
| Shirts, boys' ... ... ... | — | 18 |

If these lists are carefully compared, it will be seen what much greater demands are made upon the linen stores by the surgical than by the medical wards. The exact enumeration of the articles found necessary for the orderly discharge of duty will, it is hoped, prove useful to some who cherish the delusion that economy lies in putting the same article to half a dozen uses, and in devising perpetual makeshifts.

* "The Matron: her Duties and Responsibilities." (Scientific Press.)

